# Astigmatic changes after Descemet membrane endothelial keratoplasty (DMEK) in decompensated penetrating keratoplasty grafts

**DOI:** 10.1111/aos.70081

**Published:** 2026-02-11

**Authors:** Florian Thomas Steinberg, Jithmi Weliwitage, Marie von Berg, Silvia Schrittenlocher, Simona Schlereth, Claus Cursiefen, Björn Bachmann

**Affiliations:** ^1^ Department of Ophthalmology Medical Faculty and University Hospital of Cologne Cologne Germany; ^2^ Institute of Medical Statistics and Computational Biology (IMSB) Faculty of Medicine, University of Cologne Cologne Germany; ^3^ Medical Faculty and University Hospital of Cologne Cologne Germany

**Keywords:** astigmatism, Descemet membrane endothelial keratoplasty, penetrating keratoplasty, toric intraocular lens

## Abstract

**Purpose:**

To evaluate the surgery‐induced changes of astigmatism after Descemet membrane endothelial keratoplasty (DMEK) in eyes with failed previous penetrating keratoplasty (PK).

**Design:**

Retrospective, interventional cohort study based on prospective DMEK database.

**Methods:**

Evaluation of 15 eyes after DMEK surgery for endothelial decompensation after PK. Astigmatic changes before decompensation and after DMEK surgery were compared using anterior and posterior keratometry and True Net Power (TNP) in corneal tomography (Pentacam, Oculus). The differences in magnitude of astigmatism and axis of steep meridian were calculated by algebraic and vectorial methods, and significant differences to specified thresholds were determined by the non‐parametric Wilcoxon rank test.

**Results:**

Surgically induced changes (SIC) in magnitude of astigmatism at anterior surface, posterior surface and TNP were −1.4dpt ± 3.7, −0.2dpt ± 0.8 and −1.3dpt ± 3.9, respectively. No significant changes in astigmatism and axis difference by algebraic method in comparison with a hypothetical median of 0dpt and 10° were observed. Calculation of astigmatism difference by vectorial method showed a significant deviation in anterior surface (observed median = 4.4dpt, *p* < 0.001), posterior surface (observed median 1.0dpt, *p* = 0.012) and TNP (observed median = 3.6dpt, p < 0.001).

**Conclusion:**

Even if no significant change in magnitude and axis of astigmatism to acceptable thresholds could be shown after DMEK in failed PK using the algebraic method, there is a significant and relevant change in magnitude of astigmatism using the vectorial calculation. Based on these results, caution is advised when using toric intraocular lenses in PK eyes, as astigmatic change is expected when performing DMEK on eyes with failed PK.

## 1 INTRODUCTION

Penetrating keratoplasty (PK), first performed in 1905 (Dick et al., 2026; Zirm, 1989), was for a long time the method of choice for repeat PK in cases of graft failure. A common cause of graft failure after PK is endothelial decompensation (Hos et al., 2017). In such cases, if the corneal stroma shows no opacities beyond oedema and the patient was satisfied with the graft function prior to decompensation, endothelial keratoplasty can now be considered a standard treatment option. Descemet membrane endothelial keratoplasty (DMEK) and Descemet stripping automated endothelial keratoplasty (DSAEK) (Simons et al., 2023) have become widely accepted for endothelial disorders. Currently, the most common indications for DMEK are Fuchs' endothelial corneal dystrophy (FECD) and pseudophakic bullous keratopathy (Cursiefen & Kruse, 2010; Flockerzi et al., 2024). DMEK has replaced PK for most endothelial diseases because PK has a higher graft rejection rate (Hos et al., 2019; Woo et al., 2019), a longer postoperative recovery time (Maier et al., 2013) and a more significant change in corneal topography (Crawford et al., 1986; Dietrich et al., 2011; Riddle Jr. et al., 1998). DMEK enables an increased vision with low transplant failure and rejection rates (Spaniol et al., 2023). Also, no negative impact on peripapillary nerve fibre layer thickness could be shown between DMEK and phacoemulsification, despite known fluctuations in intraocular pressure in the early postoperative period after DMEK (Madsen et al., 2024). Compared to repeat PK, the risk of complications appears to be lower and the visual recovery time significantly shorter (Hos et al., 2021).

Meanwhile, PK is frequently associated with high astigmatism. Different patient‐related factors, surgical factors and transplant‐related factors influence postoperative astigmatism after PK. Some studies emphasize the importance of preoperative underlying diagnosis (e.g. keratoconus) as a factor influencing the degree of postoperative astigmatism (Yilmaz et al., 2007). Other studies show that surgical factors have a greater influence than preoperative diagnosis (Claesson et al., 2002). There is a lack of clear data on the influence of various factors on postoperative astigmatism, partly due to the different surgical techniques used. Addressing post‐PK astigmatism, management differs if sutures are still present or if sutures already have been removed (Fares et al., 2012). Even though irregular astigmatism is often present after PK, a regular astigmatic component can also be detected in some cases. Best option for correction are typically stable contact lenses, but these are not tolerated by every patient. Surgical options include arcuate incisions (Bohringer et al., 2016), compression sutures (Chang et al., 2003), refractive laser surgery (Bandeira et al., 2018) and the implantation of toric IOLs (Wade et al., 2014; Wan et al., 2022).

Theoretically, the decision to implant a toric intraocular lens (IOL) in patients with astigmatism after PK can be made at two distinct time points: one option is to implant the toric IOL during cataract surgery while the PK graft is still clear and corneal astigmatism is stable—that is, before any signs of endothelial decompensation occur. Alternatively, the decision can be made in the setting of graft failure, in combination with a planned DMEK procedure (Triple‐DMEK, either in phakic or pseudophakic eyes) (Chaurasia et al., 2014; Laaser et al., 2012; Romano et al., 2024). By combining the interventions, the number of operations and the cumulative risk of endophthalmitis can be reduced. In addition, DMEK favours accelerated cataract development due to surgical manipulation of the anterior chamber or the inserted gas tamponade (Moshiri et al., 2021; Price et al., 2010; Price & Price Jr., 2004) so that cataract surgery is often necessary one to two years after DMEK (Burkhart et al., 2014; Gundlach, Maier, Riechardt, et al., 2015; Gundlach, Maier, Tsangaridou, et al., 2015; Price et al., 2010). Also, endothelial cell loss occurs during phacoemulsification (Liesegang et al., 1984; Moshirfar et al., 2022; Musa et al., 2013), so that damage to the already transplanted corneal lamella can be expected in a two‐staged procedure with secondary cataract surgery. On the contrary, precise calculation of the intraocular lens is more difficult if the cornea is already decompensated. A hyperopic shift in refraction is known to occur after DMEK (Price Jr. & Price, 2017). Moreover, the accurate assessment of corneal astigmatism is limited by stromal oedema and surface irregularities, which can compromise the reliability of biometry and keratometry. To improve refractive planning under these conditions, historical keratometric values obtained prior to PK graft decompensation may serve as helpful reference. However, to date, no studies have investigated the influence of DMEK on astigmatism in a PK eye (Alnawaiseh et al., 2017; Yokogawa et al., 2017).

The aim of this study therefore was to evaluate whether a significant change in corneal astigmatism occurs in DMEK after PK compared with the situation before PK decompensation. The results could help to decide whether and when the implantation of a toric intraocular lens (IOL) in the capsular bag—either prior to decompensation of the PK graft or as part of a Triple‐DMEK procedure—represents a meaningful therapeutic approach considering that alternative strategies to correct corneal astigmatism after PK exist, such as toric add‐on lenses.

## 2 METHODS

In this retrospective interventional cohort study based on the prospective Cologne DMEK database (Schrittenlocher et al., 2017), all patients of the Department for Ophthalmology of the University Hospital of Cologne who underwent DMEK in the period from 1 July 2011 to 31 August 2021 were included. The standardized surgical procedure (Bhogal et al., 2025) and postoperative follow‐up have already been explained in previous studies (Bachmann et al., 2010; Schrittenlocher et al., 2020). The following points were defined as inclusion criteria:

- A PK had to have been performed previously on the operated eyes.
- Pentacam data must be available before and after DMEK.
- The cornea must not already be decompensated at the time of preoperative corneal Pentacam examination in order to eliminate massive corneal deswelling as an influencing factor. To assess the absence of corneal oedema, both the description of transparency from the slit lamp examination in the patient's file and the analysis of Scheimpflug images for a loss of parallel isopachs, thinnest point displacement and focal posterior corneal surface depression were checked (Sun et al., 2019). No pachymetric threshold was chosen as increased corneal thickness with a central pachymetry of 608 ± 75 μm is already detected after successful penetrating keratoplasty (Kus et al., 1999). Selecting a threshold value based on the typical pachymetry of 550 μm would have resulted in incorrect exclusion of Scheimpflug images.
- All PK sutures must have been removed at the time of Pentacam examination, as these would have had a significant influence on astigmatism.
- Regular astigmatism should be visible in the corneal topography data before DMEK.

### 2.1 Data aggregation

This is a retrospective analysis of DMEK patients from our ‘Cologne DMEK database’ (IRB approval number 14–373). REDCap (‘Research Electronic Data capture’) was used as data collection tool. All guidelines of the Declaration of Helsinki were observed.

**FIGURE 1:**
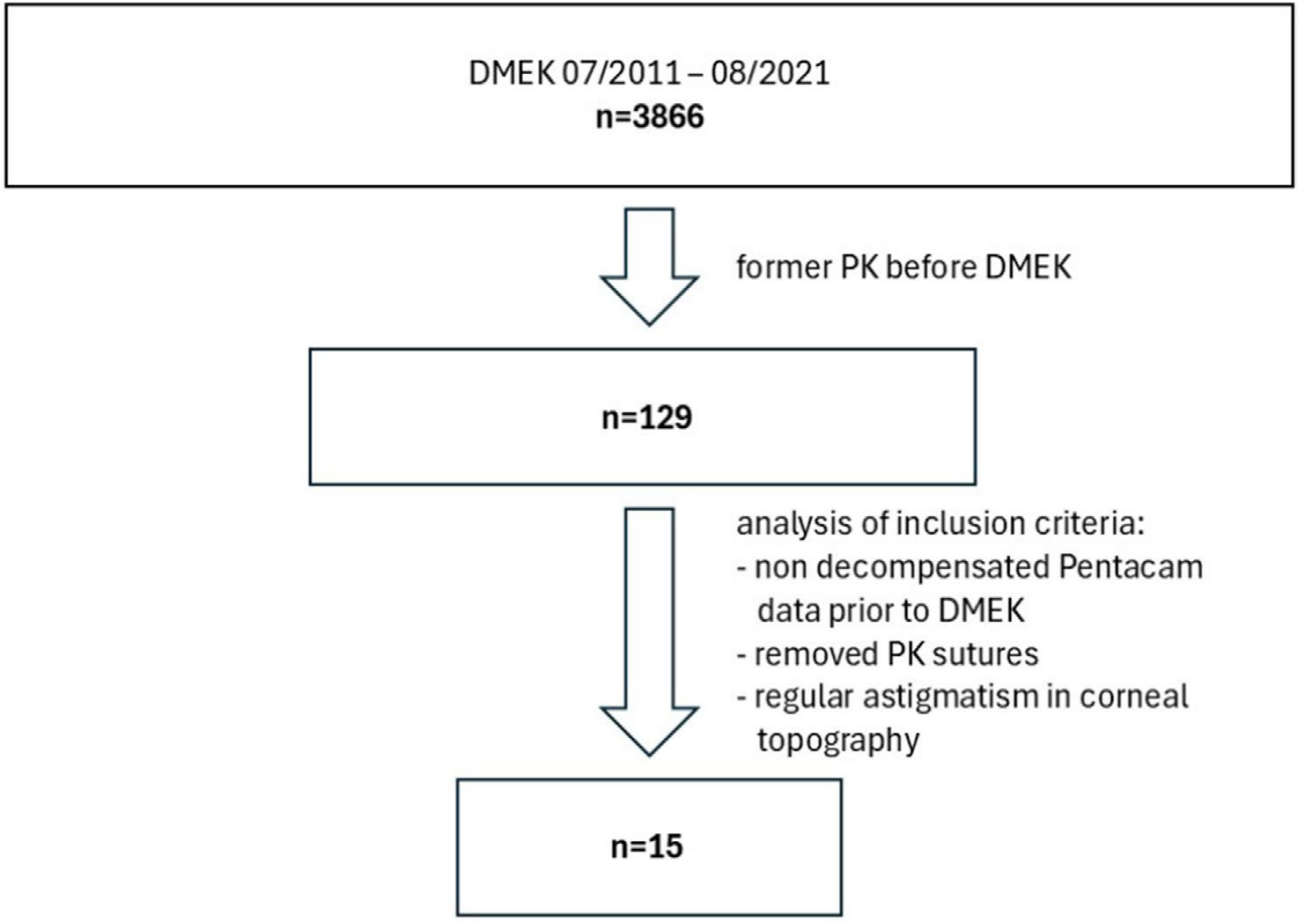
Case number trend after analysis of inclusion criteria. DMEK, Descemet membrane endothelial keratoplasty; PK, penetrating keratoplasty.

The Cologne DMEK Database was reviewed, and the patients were checked for inclusion criteria. A total of 3866 patients were operated on during the above‐mentioned period, of whom 15 patients were ultimately included in this study. A flow chart of the selection process is shown in Figure 1.

### 2.2 Astigmatism evaluation

The Pentacam HR (Oculus GmbH, Wetzlar, Germany) was used to generate corneal topographic maps. Central corneal thickness, mean keratometry values, the steepest and flattest meridians and the axis of the steepest meridian were recorded. The keratometry values of the corneal anterior surface, the posterior surface and the ‘true net power’ as a surrogate for the total corneal refractive power are analysed separately. In analogy to previous analyses, the keratometry values refer to the central 15° (3 mm ring), centred on the apex (Alnawaiseh et al., 2017).

Astigmatism is classified according to the axis of the steepest meridian (Alnawaiseh et al., 2017; Kamiya et al., 2015; Nemeth et al., 2014). The following thresholds apply to the corneal front surface and the true net power:

- ‘with‐the‐rule’ (WTR): 60–120°
- ‘against‐the‐rule’ (ATR): 0–30° and 150–180°
- ‘oblique’: axis positions outside the above‐mentioned limits

Due to the negative refractive power of the corneal back surface, the classification is opposite to that of the corneal front surface (Alnawaiseh et al., 2017).

### 2.3 Astigmatism calculation

Astigmatism is calculated by definition as the difference between the steepest and flattest meridians.

**FIGURE 2:**
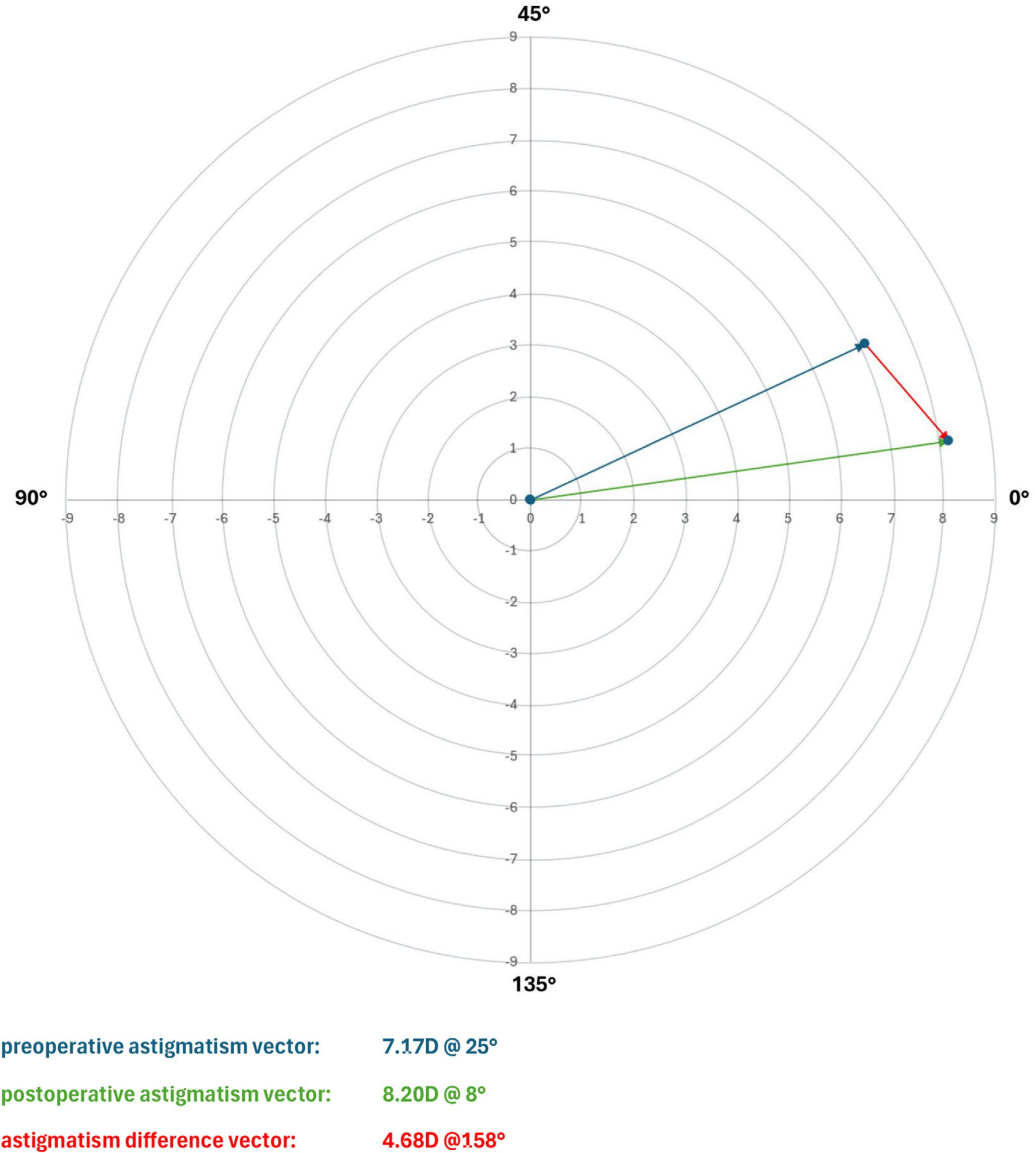
Schematic model of vector calculation. Representation of the difference vector using the pre‐ and postoperative vector. The absolute value of the difference vector represents the amount of the vectorial astigmatism difference. The direction of the difference vector indicates the vectorial axial difference.

The difference in astigmatism between the preoperative baseline and postoperative value (‘surgically induced astigmatism’, SIA) can be calculated by two different methods (Alnawaiseh et al., 2017; Yokogawa et al., 2016):

1. Algebraic method: arithmetic subtraction of the preoperative astigmatism from the postoperative astigmatism. This method does not consider any change in the steepest meridians axis caused by surgery.
2. Vector method: Astigmatism with axis position is regarded as a vector. The amount of astigmatism is represented by the length of the vector and the direction by the axis position. The length of the difference vector between the preoperative and postoperative vectors represents SIA. A schematic model of the vector calculation is shown in Figure 2.

The difference vector is calculated according to the procedure described by Holladay et al. (2001). Consequently, the first step is to convert the polar Pentacam data into Cartesian values according to the laws of trigonometry:

$$X\;\text{value}=\text{astigmatism}\times \cos\;\left(2^{*}\;\text{axis}\;\left(\text{steep}\right)\right),$$

$$Y\;\text{value}=\text{astigmatism}\times \sin\;\left(2^{*}\;\text{axis}\;\left(\text{steep}\right)\right).$$

The length of the difference vector is then calculated using Pythagoras' theorem:

$$A_{\text{diff}}=\sqrt{{\left(x_{\text{post}}-x_{\mathrm{pre}}\right)}^{2}\text{Astig}+{\left(y_{\text{post}}-y_{\mathrm{pre}}\right)}^{2}}$$

(*A*_*diff*_ = magnitude of the difference vector).

The axis of the difference vector can also be calculated in a similar way using trigonometry:

$$\alpha_{\text{diff}}=\frac{1}{2}\times \tan^{-1}\left(\frac{y_{\text{post}}-y_{\mathrm{pre}}}{x_{\text{post}}-y_{\mathrm{pre}}}\right)$$

Finally, the axis position of the difference vector had to be converted back to polar values to enable a valid interpretation. The magnitude and axis position of the difference vector can then be represented in a polar coordinate system (‘double‐angle plot’).

Since both the difference in astigmatism and in axis are considered, the common term ‘surgically induced astigmatism’ (SIA) is replaced by ‘surgically induced change’ (SIC) in this study.

### 2.4 Statistical evaluation

The software SPPS Version 28.0.1.0 (SPSS, Inc., Chicago, IL) was used for statistical analysis.

For descriptive statistics, the pre‐ (before DMEK) and postoperative (after DMEK) central corneal thickness, the mean keratometry readings, the degree of astigmatism and the axis of the steepest meridian are each reported in relation to the corneal anterior surface, posterior surface and true net power using the mean ± standard deviation and 95% confidence interval.

For SIC, the difference between pre‐ and postoperative central corneal thickness and mean keratometry readings is calculated as the mean ± standard deviation and confidence interval. The SIC for the amount of astigmatism and the axis of the steepest meridian is calculated for the algebraic and vectorial difference using the methods mentioned above and is given as the mean ± standard deviation and 95% confidence interval. Grouped box plots are used for the graphical representation of the SIC. Box plots display the 1st and 3rd quartiles (bottom and top lines of the box), the median (middle line) and the whiskers show the maximum and minimum values with the exceptions of outliers (circles, distance more than 1.5 times the interquartile range) and extremes (asterisks, distance more than 3 times the interquartile range).

Due to the number of cases, non‐parametric tests are used for the significance analysis. The astigmatism difference based on the algebraic and vector methods and the axis difference based on the algebraic method are tested using the Wilcoxon sign rank test. For those three parameters, three different hypothetical medians are used. The astigmatism difference according to the algebraic method has either positive or negative values for each individual eye, so ‘0D’ as the hypothetical median was chosen. The amount of astigmatism difference based on the vector method and the axis difference of the steepest meridians are each the mathematical absolute value without the possibility of negative values. Therefore, a tolerable deviation from the optimal difference of 0D or 0° was chosen as hypothetical median. A tolerable deviation of 0.5D was chosen for the astigmatism difference using the vector method, and a tolerable deviation of 10° (Oshika et al., 2018) was chosen for the axis difference using the algebraic method. P‐values <0.05 are defined as the significance level. A significance level ≥0.05 supports retention of the null hypothesis, in which there is no statistically significant SIC. The change in the vectorial axis difference is not statistically examined due to its lack of clinical relevance.

To verify the statistical robustness of the results, the Wilcoxon sign rank test excluding those outliers that are >1.5 times the interquartile range from the upper or lower quartile is additionally performed for significance analysis. For graphical representation of outliers in a polar coordinate system, the vector data are displayed in a double‐angle plot. In the double‐angle plot, the amount of astigmatism or the vectorial astigmatism difference is represented by the distance to the centre and the axis of the steepest meridian or the vectorial axis difference is represented by the direction. The individual data for each eye is reflected by single points; a square represents the aggregated value for all eyes.

## 3 RESULTS

A total of 15 eyes of 15 patients who underwent DMEK surgery after a PK during the specified period met the inclusion criteria of this study. Mean age at DMEK was 65.5 ± 11.8 years, and 9 of 15 patients (60%) were female. The most common indication for PK was FECD (4 out of 15 patients), followed by keratoconus, congenital glaucoma, lattice corneal dystrophy (3 out of 15 patients each) and herpes keratopathy (2 out of 15 patients). Due to the retrospective nature of the study, the exact cause of endothelial graft failure cannot be determined in many cases. Most commonly, Guttae were found on the graft (3 out of 15 patients), followed by an immunological graft rejection (2 out of 15 patients). In two‐thirds of cases, the medical records did not reveal a clear cause for the graft failure, so secondary graft failure due to graft aging or a past graft rejection must be assumed. The mean time interval between PK and DMEK was 14.4 ± 9.1 years. Epidemiologic and baseline data are presented in Table 1. The different time intervals between the operations and the Scheimpflug images are shown in Table 2.

**TABLE 1:** Epidemiologic and baseline data.

		*n*	(%)	mean	Standard deviation	Median	IQR (1. to 3. quartile)
Total		15					
Sex	Female	9	60.0				
	Male	6	40.0				
Age (years)				65.53	±11.81	67.0	18.0 (58.0–76.0)
Indication for PK	Fuchs endothelial dystrophy	4	26.7				
	Keratoconus	3	20.0				
	Congenital glaucoma	3	20.0				
	Herpes keratopathy	2	13.3				
	Latticed corneal dystrophy	3	20.0				
Indication for DMEK	Edema with Guttae on transplant	3	20.0				
	Immune reaction	2	13.3				
	Edema in transplant failure	10	66.6				
Operated eye	Right eye	8	53.3				
	Left eye	7	46.7				
Time interval between PK and DMEK (years)				14.39	±9.14	12.4	12.2 (7.4–19.6)
Pachymetry preoperative (μm)				575.13	±72.51	612.0	105.0 (520.0–625.0)

Abbreviations: DMEK, Descemet membrane endothelial keratoplasty; IQR, interquartile range; PK, penetrating keratoplasty.

**TABLE 2:** Representation of the different time intervals between the operations and the measurement points of the Scheimpflug images.

	*n*	Mean ± standard deviation (month)
Time between DMEK and PK	14	172.0 ± 109.5
Time between PK and preoperative Scheimpflug	14	154.4 ± 117.2
Time between preoperative Scheimpflug and DMEK	15	16.2 ± 10.9
Time between DMEK and postoperative Scheimpflug	15	23.4 ± 19.8

*Note*: *n* = count. In one case of an externally performed PK, the time point of the PK cannot be traced back clearly due to the retrospective nature of this study, so that case count is reduced to *n* = 14.

**FIGURE 3:**
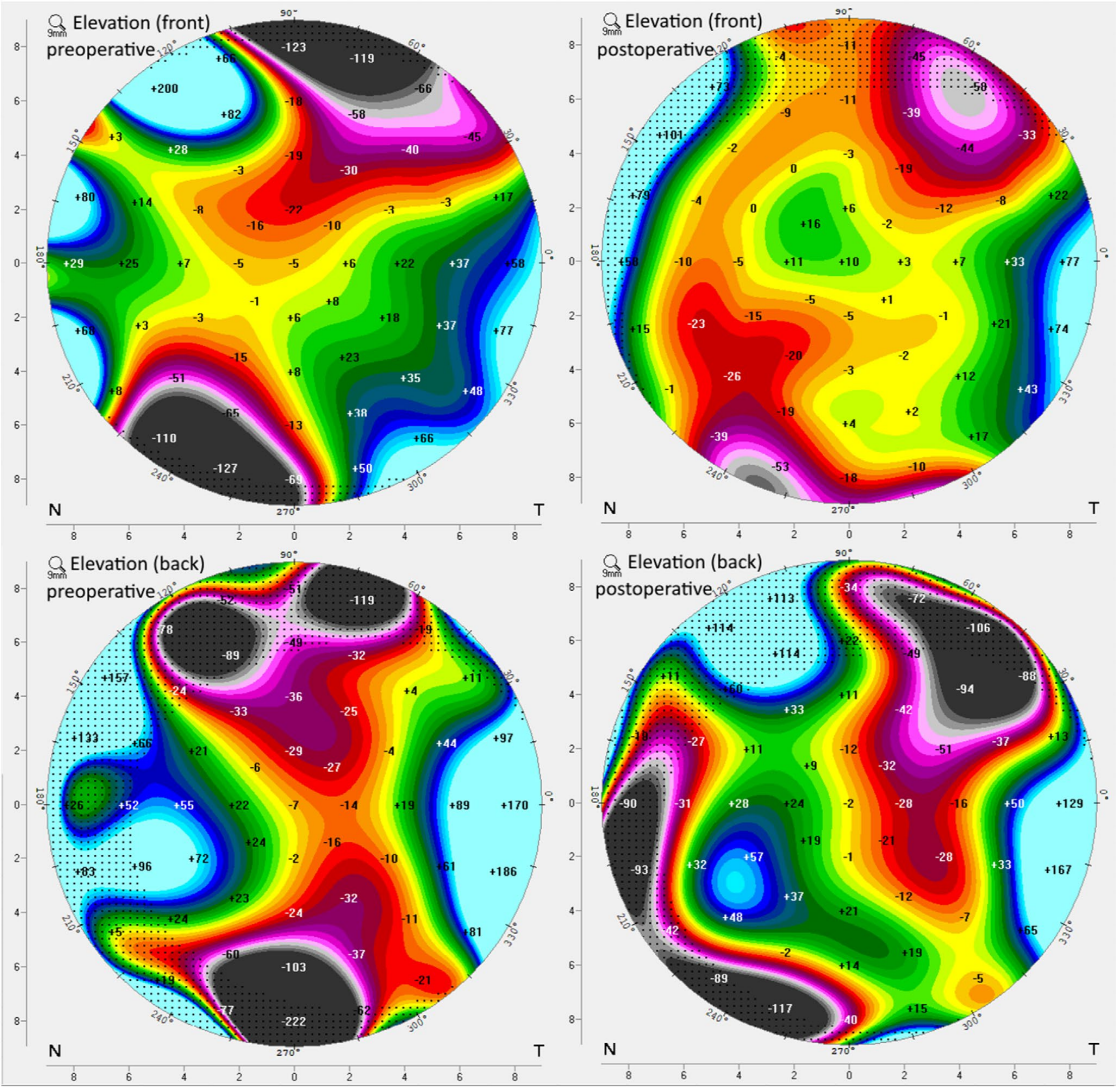
Grouped boxplots of difference in astigmatism and axis between preoperative value before corneal decompensation and after DMEK calculated by algebraic and vectorial method. Height map comparison of corneal front and back surface 11 months before and 5 years after DMEK. The best‐fitted sphere (BFS) was selected as a reference in this illustration. In this case, there is a clear elevation in the centre of the corneal front surface. The increased steepness of the corneal front surface results in an increase in mean keratometry and thus a myopic shift. There is a clear increase in mean keratometry from 41.6D (preoperative) to 49.3D (postoperative) with a reduction in astigmatism from 5.2D (preoperative) to 3.4D (postoperative).

**FIGURE 4:**
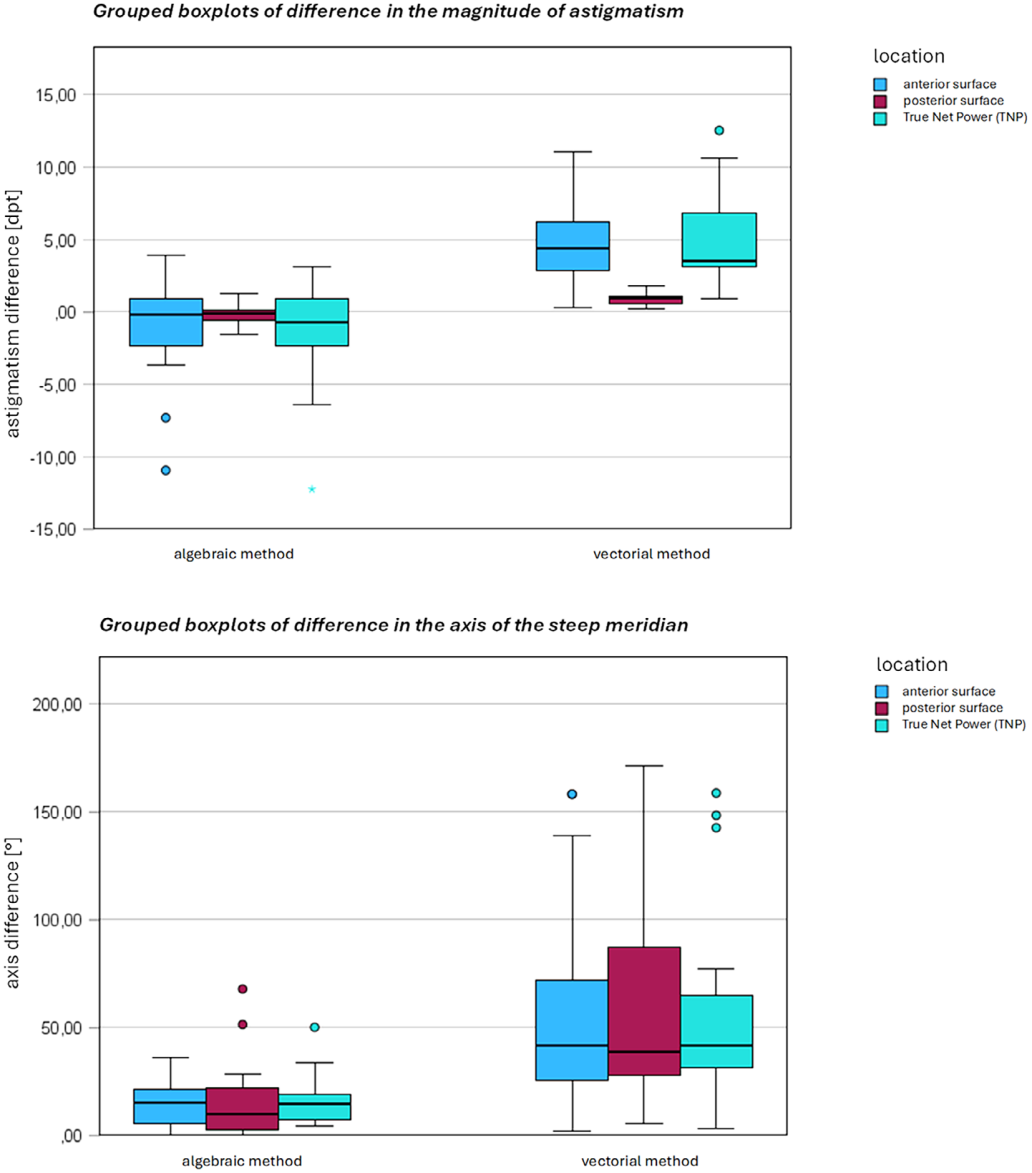
Double‐angle plots for magnitude and axis of astigmatism in anterior surface, posterior surface and True Net Power. Representation of astigmatism and axis difference via Boxplot using the algebraic and vectorial method. The difference at the corneal anterior surface, posterior surface and True Net Power (TNP) are indicated by different colours.

The preoperative mean keratometry readings of the corneal anterior surface, posterior surface and true net power were 46.8 ± 3.9D, −6.9 ± 0.8D and 44.6 ± 4.7D, respectively. Postoperatively, SIC in mean keratometry readings was 1.0 ± 3.1D, 0.1 ± 0.5D and 1.7 ± 4.5D, respectively. An exemplary height map comparison illustrating the Kmean increase is shown in Figure 3. Preoperative pachymetry at the time point of no endothelial decompensation was 575 ± 73 μm. Postoperatively after DMEK, there was a decrease in central corneal thickness of −82 ± 76 μm to a level of 492 ± 39 μm. A graphical representation of the algebraic and vectorial astigmatism and axis difference by boxplots is shown in Figure 4.

### 3.1 Change in astigmatism

Preoperatively, there was a mean astigmatism with an axis of the steepest meridian of 8.8 ± 4.4D at 84 ± 49° on the corneal anterior surface, 1.4 ± 0.7D at 85 ± 53° on the corneal posterior surface and 8.6 ± 4.6D at 86 ± 49° in true net power (Table 3).

**TABLE 3:** Pre‐ and postoperative keratometric data and surgically induced change.

		Preoperative	Postoperative	Surgically induced change	
Central corneal thickness (μm)		575.1 ± 72.5 (CI: 535.0 to 615.3)	492.8 ± 39.3 (CI: 471.0 to 514.6)		−82.3 ± 76.4 (CI: −124.7 to −40.0)
Anterior surface	Mean keratometry (D)	46.8 ± 3.9 (CI: 44.7 to 48.9)	47.8 ± 4.1 (CI: 45.5 to 50.1)		1.0 ± 3.1 (CI: −0.7 to 2.7)
	Magnitude of astigmatism (D)	8.8 ± 4.4 (CI: 6.3 to 11.2)	7.4 ± 4.2 (CI: 5.1 to 9.7)	Algebraic method	−1.4 ± 3.7 (CI: −3.5 to 0,7)
				Vectorial method	4.6 ± 2.7 (CI: 3.1 to 6.2)
	Axis of steep meridian (°)	84.3 ± 49.0 (CI:57.2 to 111.4)	85.4 ± 56.8 (CI: 54.0 to 116.9)	Algebraic method	14.9 ± 11.4 (CI: 8.6 to 21.2)
				Vectorial method	58.8 ± 53.1 (CI: 29.4 to 88.2)
Posterior surface	Mean keratometry (D)	−6.9 ± 0.8 (CI: −7.4 to −6,5)	−6.8 ± 0.6 (CI: −7.3 to 6.6)		−0.1 ± 0.5 (CI: −0.3 to 0.2)
	Magnitude of astigmatism (D)	1.4 ± 0.7 (CI: 1.0 to 1.8)	1.2 ± 0.8 (CI: 0.8 to 1.7)	Algebraic method	−0.2 ± 0.8 (CI: −0.6 to 0.3)
				Vectorial method	0.9 ± 0.5 (CI: 0.6 to 1.2)
	Axis of steep meridian (°)	85.3 ± 52.9 (CI: 56.0 to 114.6)	94.7 ± 49.3 (CI: 67.4 to 122.0)	Algebraic method	17.7 ± 19.4 (CI: 6.9 to 28.4)
				Vectorial method	63.8 ± 53.5 (CI: 34.2 to 93.4)
True net power	Mean keratometry (D)	44.6 ± 4.7 (CI: 42.0 to 47.2)	46.3 ± 4.2 (CI: 44.0 to 48.6)		1.65 ± 4.5 (CI: −0.8 to 4.1)
	Magnitude of astigmatism (D)	8.6 ± 4.6 (CI: 6.0 to 11.1)	7.3 ± 4.0 (CI: 5.1 to 9.4)	Algebraic method	−1.3 ± 3.9 (CI: −3.5 to 0.8)
				Vectorial method	5.0 ± 3.3 (CI: 3.3 to 6.8)
	Axis of steep meridian (°)	86.0 ± 49.3 (CI: 58.6 to 113.3)	76.4 ± 52.8 (CI: 47.2 to 105.7)	Algebraic method	16.4 ± 12.7 (CI: 9.4 to 23.5)
				Vectorial method	59.4 ± 50.4 (CI: 31.4 to 87.3)

Abbreviation: CI, 95% confidence interval.

Using the algebraic method, a SIC of −1.4 ± 3.7D (95% CI ‐3.5 to 0.7) was found on the anterior surface, 0.2 ± 0.8D (95% CI ‐0.3 to 0.6) on the posterior surface and − 1.3 ± 3.9D (96% CI −3.5 to 0.8) in true net power. The Wilcoxon sign rank test showed an observed median of −0.2D (*p* = 0.233 to a hypothetical median of 0D) on the anterior surface, −0.1D (*p* = 0.495 to a hypothetical median of 0D) on the posterior surface and −0.7D (*p* = 0.334 to a hypothetical median of 0D) in true net power (Table 4). In the sensitivity analysis after exclusion of outliers, similar values were found with a median of 0.1D (*n* = 13, *p* = 0.650) on the anterior surface and −0.3D (*n* = 14, *p* = 0.551) in the true net power. No outliers were found on the posterior corneal surface. The algebraic astigmatism difference shows no significant difference from the hypothetical median of 0D on all three surfaces examined by Wilcoxon sign rank test.

**TABLE 4:** Wilcoxon sign rank test and sensitivity analysis except outliers.

			With outliers				Except outliers			
			*n*	Hypothetical median	Observed median	*p*‐value	*n*	Hypothetical median	Observed median	*p*‐value
Magnitude of astigmatism (dpt)	Algebraic method	Anterior surface	15	0.0	−0.2	0.233	13	0.0	0.1	0.650
		Posterior surface	15	0.0	−0.1	0.495	15	0.0	−0.1	0.495
		True Net Power	15	0.0	−0.7	0.334	14	0.0	−0.3	0,551
	Vectorial method	Anterior surface	15	0.5	4.4	<0.001	15	0.5	4.4	<0.001
		Posterior surface	15	0.5	1.0	0.012	15	0.5	1.0	0.012
		True Net Power	15	0.5	3.6	<0.001	14	0,5	3,5	<0.001
Axis of steep meridian (°)	Algebraic method	Anterior surface	15	10.0	15.4	0.164	15	10.0	15.4	0.164
		Posterior surface	15	10.0	10.0	0.272	13	10.0	9.3	0.754
		True Net Power	15	10.0	14.9	0.094	14	10.0	11.7	0.167

*Note*: *n* = count.

Using the vector method, a SIC of 4.6 ± 2.7D (95% CI −3.1 to 6.2) was found on the anterior surface, 0.9 ± 0.5D (95% CI −0.6 to 1.2) on the posterior surface and 5.0 ± 3.3D (96% CI 3.3 to 6.8) in true net power. The significance analysis using the Wilcoxon sign rank test showed an observed median of 4.4D (*p* < 0.001 to a hypothetical median of 0.5D) on the anterior surface, 1.0D (*p* = 0.012 to a hypothetical median of 0.5D) on the posterior surface and 3.6D (*p* < 0.001 to a hypothetical median of 0.5D) in the true net power. There was only one outlier in true net power, which did not significantly influence the result in the sensitivity analysis (median = 3.5D, *p* < 0.001 to a hypothetical median of 0.5D). In the Wilcoxon sign rank test, the null hypothesis is rejected for the vector astigmatism difference on all three surfaces.

**FIGURE 5:**
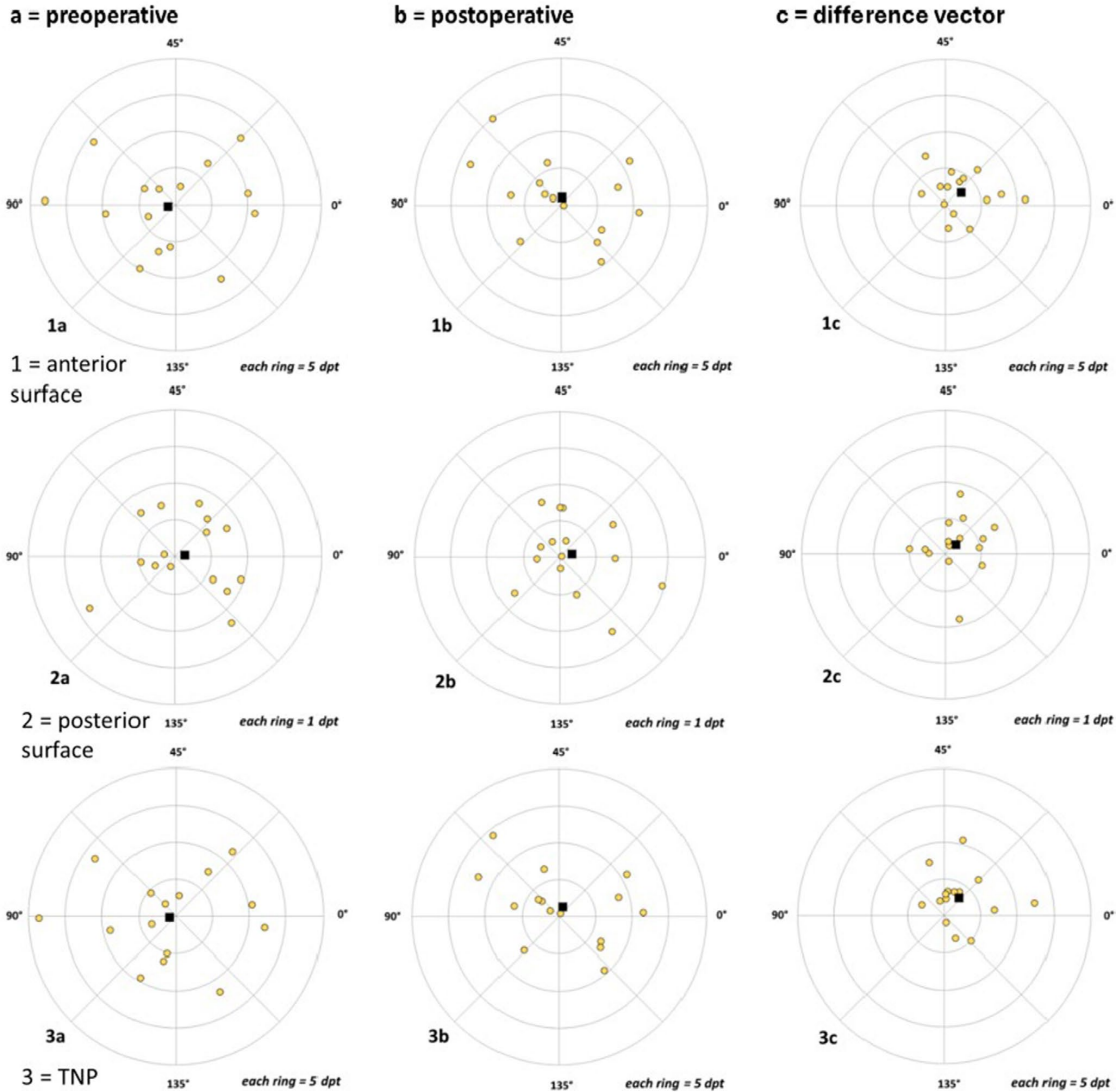
Double‐angle plots for magnitude and axis of astigmatism in anterior surface, posterior surface and True Net Power. Display of double‐angle plots in which the distance to the centre represents the amount of astigmatism and the direction represents the axis of the steepest meridian. Each dot corresponds to a patient's data point, while the square represents the centroid value. The top row shows the double‐angle plots for the corneal anterior surface (1), the middle row shows the corneal posterior surface (2), and the bottom row shows the True Net Power (3). The columns represent the preoperative values (a), the postoperative values (b) and the difference vector (c).

The double‐angle plot shows a vector astigmatism difference on the anterior surface of >5D in 5 cases, including one case >10D (Figure 5). The quality of the topometric data was checked consecutively.

### 3.2 Change in axis

The SIC of the axis of the steepest meridian was only examined regarding the algebraic difference. A mean axis difference of 15 ± 11° (95% CI 9 to 21) was found on the anterior surface, 18 ± 19° (95% CI 7 to 28) on the posterior surface and 16 ± 13° (95% CI 9 to 24) in the true net power. The Wilcoxon sign rank test showed a median of 15.4° (*p* = 0.164) on the front surface, 10.0° (*p* = 0.272) on the rear surface and 14.9° (*p* = 0.094) in the true net power, indicating that the hypothetical median of 10° as selected threshold value was exceeded. However, the necessary significance level to reject the null hypothesis was not reached. The sensitivity analysis excluding outliers (*n* = 2 outliers on the posterior surface, *n* = 1 outlier in true net power) changes the results just marginally.

## 4 DISCUSSION

In many patients with corneal endothelial decompensation, DMEK is now combined with phacoemulsification and artificial lens implantation, known as Triple‐DMEK. A combined procedure is preferred over a two‐staged procedure for most patients as phakic DMEK promotes accelerated cataract development (Moshiri et al., 2021), and a consecutive cataract operation leads to a loss of endothelial cells of slightly more than 10% (Bamdad et al., 2018). Since most corneal surgeries change keratometry values relevant for IOL calculation, the influence of DMEK on corneal refraction in FECD has been investigated in several studies. For example, a hyperopic shift of +0.74D and +0.43D has been described for DMEK in phakic patients (Parker et al., 2012) and for Triple‐DMEK (Schoenberg et al., 2015), respectively. Hyperopia induced by DMEK is well known and should be considered when selecting lenses for Triple‐DMEK. In particular, a more pronounced preoperative corneal oedema enhances the effect of hyperopic shift (Price et al., 2009). Similarly important is the use of hydrophobic IOLs in this context to avoid IOL calcification, especially in multifocal IOLs (Schrittenlocher et al., 2017).

Due to the stability of the refractive indices, particularly in the Triple‐DMEK procedure, the use of toric IOLs in the context of a Triple‐DMEK has already been evaluated. In various studies of DMEK on non‐PK (naïve) cornea, a vectorial astigmatism difference of 0.26–0.77D (Alnawaiseh et al., 2017; Yokogawa et al., 2016) and an arithmetic astigmatism difference of 0.16–0.59D (Alnawaiseh et al., 2017; Machalinska et al., 2021; Yokogawa et al., 2016) were found at the anterior corneal surface. Based on the low astigmatism difference, patients have already been successfully treated with toric intraocular lens implantation for Triple‐DMEK (Hamon et al., 2022; Trindade et al., 2021; Yokogawa et al., 2017). Other authors rather point out a lack of astigmatism predictability after DMEK (Shajari et al., 2019).

Whether astigmatism stability is also present after DMEK in an eye that has undergone prior PK surgery has not yet been investigated. Assuming that DMEK restores the conditions that existed prior to endothelial decompensation in such patients, historic keratometry from periods before PK decompensation could be used for IOL calculation. The influence of the anterior and the posterior surface keratometry by DMEK in FECD patients has already been investigated (Diener et al., 2020; Rangu et al., 2024). Our study is the first investigation regarding keratometric changes following DMEK in patients with decompensated PK grafts. In statistical significance analysis, there is no significant deviation from the hypothetical median of 0D in astigmatism difference and 10° in axis difference on the corneal surface by algebraic method. Otherwise, using the vectorial method, a mean SIC of 4.6D is found on the corneal anterior surface. While the algebraic astigmatism difference provides a fast and rough assessment of astigmatism change in clinical practice, the vectorial astigmatism difference considers both the change in height and the change in axis. The vectorial astigmatism difference can therefore quantify the actual optical effect better than the algebraic difference. In addition, the double‐angle plot shows a high scatter width in the vectorial astigmatism difference despite a good centroid value. Although some patients have only a small SIC, others show unpredictability of astigmatism change that would lead to a non‐valid calculation of a toric intraocular lens. In particular, the low tolerance of residual refractive errors when using premium lenses must be considered (Trindade et al., 2021).

These results contrast with previous studies, which have shown relevant changes in posterior corneal surface with just marginal changes in the anterior corneal surface with DMEK on naïve corneas (Ham et al., 2011). Thera are various possible causes for the results deviation of this study from the previous refractive results with DMEK on naïve corneas. After PK, a high astigmatism even after removing the sutures can occur. This study shows a preoperative mean astigmatism of 8.8D, whereas in previous studies on DMEK on naïve corneas, the astigmatism was in the range of 1–2D (Alnawaiseh et al., 2017; Machalinska et al., 2021; Yokogawa et al., 2016). The higher the astigmatism before PK decompensation, the higher the magnitude of the difference vector compared with a low preoperative astigmatism. Also, the higher the anterior and total corneal astigmatism, the more pronounced the role of the corneal posterior surface for refractive changes (Alnawaiseh et al., 2017), which is significantly influenced by DMEK. In addition, it could be shown that not only the height of the astigmatism plays a role, but also the astigmatism difference in different zones is relevant. A reduction in postoperative corneal astigmatism, as also observed in this study, was described in particular in those eyes with a high astigmatism difference between the 3 mm and 5 mm zones (Shajari et al., 2019). Preoperative corneal swelling could play an additional role. One inclusion criterion for this study was the availability of keratometry data at a time point without clinical corneal oedema. The central corneal thickness before graft decompensation was found to be normal, averaging 575 μm. After successful DMEK, however, there was a significant reduction in corneal thickness to 493 μm. The deswelling of the PK by DMEK could play an additional role in the significant postoperative astigmatism difference and axial deviation. Even though almost 90% of all potential cases were excluded based on the inclusion criteria, it appears that in some cases subclinical oedema that was already present during the Scheimpflug examination was not detected. It must therefore be discussed that the criteria for subclinical oedema as they apply to FED (non‐parallel isopachs, thinnest point displacement and focal corneal posterior surface elevation) (Sun et al., 2019) cannot be transferred to subclinical oedema in PK. Further aspects to consider when planning a DMEK for repair of failed previous PK graft are the slightly increased risk of endothelial immune reactions (28) compared with ‘normal’ DMEK (Hos et al., 2017) and the increased risk for IOL calcification if hydrophilic lenses are in place (Schrittenlocher et al., 2017).

Furthermore, a change in corneal curvature from the time of the preoperative Scheimpflug image to the DMEK due to increasing corneal oedema should be considered. Even though the aim of DMEK is to restore the preoperative non‐decompensated state of the cornea, persistent corneal fibrosis has been shown to cause reduced visual acuity in cases of long‐standing corneal oedema (Friedrich et al., 2025) and could thereby have an impact on corneal surface and astigmatism. At the Department for Ophthalmology of the University Hospital of Cologne, attempts are made to reduce the duration of severe corneal oedema by individually adjusting the waiting time for a corneal transplant to the degree of corneal swelling. In this study, the average duration between preoperative Pentacam and DMEK is 16 months.

In addition to the described change in astigmatism, an increase in the mean keratometry of the corneal anterior surface and in the TNP of approximately +1D was also demonstrated. This indicates an elevation of the cornea with a resulting myopic shift after DMEK on decompensated PK. This contrasts with studies following DMEK on naïve corneas, where a mild hyperopic shift of +0.5D has been described due to changes in the posterior corneal curvature (Augustin et al., 2023). In FED, there is a central decompensation with depression of the posterior corneal surface. Following DMEK for FED, elevation of the posterior surface occurs, resulting in a hyperopic shift, which does not occur with DMEK following PK.

Although a myopic shift of ‐1D after DMEK on PK has already been described (Gundlach, Maier, Riechardt, et al., 2015; Gundlach, Maier, Tsangaridou, et al., 2015), this phenomenon has not been discussed in the literature by now. One reason for this could be the biomechanical coupling of the peripheral graft to the interface, whereas the central cornea is more mobile. When the swelling is relieved by DMEK, the central cornea can bulge forward, while the periphery is mechanically fixed by the interface. Alternatively, a PK seems more susceptible to corneal scarring by long‐standing corneal oedema than naïve cornea, as the corneal fibrosis response due to the surgical trauma is higher (Torricelli et al., 2016). So, even mild corneal scarring or subepithelial haze can change the anterior surface in an unpredictable manner and can provoke, as in this study, a myopic shift. However, it must be considered that the changes in mean keratometry in our study are not significant and these results should therefore be interpreted with caution, even though they are consistent with previous studies.

The most important limitation of this study is the small number of cases and the retrospective study design. The main problem in case inclusion was the reduced number of patients with preoperative Scheimpflug images of sufficient quality without corneal oedema and without graft sutures after PK. Since successfully performed PKs without rejection or decompensation are generally managed by the outpatient sector, patients are typically referred to our centre when corneal decompensation has already occurred. Even though the Department for Ophthalmology at the University Hospital of Cologne is the largest DMEK transplant centre in Germany, only a small number of patients could be included. The limited number of cases also makes statistical analysis difficult. We attempted to mitigate the influence of outliers using non‐parametric tests and sensitivity analyses. Overall, however, the statistical significance levels should be interpreted with caution. In addition, the assessment of preoperative corneal oedema was primarily based on the clinical assessment of the cornea during the visit and pachymetry of the central cornea.

## 5 CONCLUSION

DMEK for repair of failed previous PKs results in significant astigmatism difference and axial deviation when comparing post‐DMEK values with astigmatism prior to decompensation of the PK graft. Using simultaneous toric IOLs when performing a Triple‐DMEK at the time of endothelial decompensation of PK based on keratometric data prior to decompensation of the old graft is therefore not recommended. Intracapsular toric lenses should also only be used carefully to treat astigmatism after primary PK, as astigmatism changes in the event of decompensation and subsequent DMEK. Alternative methods for astigmatism correction like arcuate incisions or the implantation of a toric Add‐on‐IOL in the ciliary sulcus after cataract surgery with a non‐toric IOL implanted in the capsular bag should be considered.

## FUNDING INFORMATION

The authors receive support by DFG SFB 1607 (www.sfb1607.de to BB, CC, FTS, SS).
